# Decomposition of Black and White Infant Mortality Rates

**DOI:** 10.1001/jamanetworkopen.2026.35321

**Published:** 2026-09-22

**Authors:** Monica J. Alexander, Rohan Alexander, Leslie Root

**Affiliations:** 1 Department of Statistical Sciences, University of Toronto, Toronto, Ontario, Canada; 2 Department of Sociology, University of Toronto, Toronto, Ontario, Canada; 3 Faculty of Information, University of Toronto, Toronto, Ontario, Canada; 4 Institute of Behavioral Science, University of Colorado Boulder, Boulder

## Abstract

**Importance:**

The infant mortality rate (IMR) of the non-Hispanic Black population in the US remains more than twice that of the non-Hispanic White population. Understanding this disparity in terms of differences in gestational age distribution, mortality risk within gestational age categories, and specific causes of death is essential for targeting interventions.

**Objective:**

To decompose the Black and White infant mortality gap into gestational age composition and mortality risk components and to identify the causes of death that may be factors in recent changes.

**Design, Setting, and Participants:**

This cross-sectional study analyzed Period Linked Birth/Infant Death Records from the US National Center for Health Statistics from January 1, 2003, through December 31, 2024. Participants included all live births to non-Hispanic Black mothers and non-Hispanic White mothers with known gestational age and plausible birth weight for gestational age.

**Exposures:**

Maternal race and ethnicity of non-Hispanic Black or non-Hispanic White.

**Main Outcomes and Measures:**

The primary measures were the composition effect (differences in gestational age distribution) and rate effect (differences in within–gestational age mortality) from a Kitagawa decomposition of the mortality difference between infants born to Black and to White mothers, overall and by specific cause of death.

**Results:**

There were 57 870 726 live births and 336 384 infant deaths in the non-Hispanic Black (141 651 [38.7%] of all deaths) and non-Hispanic White (224 733 [61.2%] of all deaths) populations between 2003 and 2024. The Black IMR declined from 13.2 (95% CI, 12.9-13.5) to 10.7 (95% CI, 10.5-11.0) deaths per 1000 live births between 2003 and 2024, while the White IMR declined from 5.6 (95% CI, 5.5-5.7) to 4.3 (95% CI, 4.2-4.4) deaths per 1000 live births. The absolute gap decreased from 7.7 to 6.0 deaths per 1000 live births between 2003 and 2019, but then increased to 6.4 (95% CI, 6.1-6.8) deaths per 1000 live births. The gestational age composition effect accounted for a majority of the difference across study years, but the rate effect increased as a share of the gap from 21% (95% CI, 18%-24%) in 2003 to 32% (95% CI, 28%-35%) in 2024. Between 2020 and 2024, the share of sudden unexpected infant death (SUID) to the gap showed the largest increase of any single cause (0.18 deaths per 1000 live births; SUID gap in 2024, 1.56 [95% CI, 1.42-1.71] deaths per 1000 live births).

**Conclusions and Relevance:**

In this cross-sectional study of IMRs, gestational age composition differences accounted for the largest share of the Black and White infant mortality gap. However, the increased share of the mortality risk component over time underscores the need for targeted interventions beyond preterm birth prevention.

## Key Points

**Question:**

How has the relative contribution of the 2 components of the Black and White infant mortality gap—gestational age composition and within–gestational age mortality risk—varied over time and for different causes of death?

**Findings:**

In this cross-sectional study of 57 870 726 live births and 366 384 infant deaths from 2003 to 2024, most of the mortality gap between infants born to Black and White mothers was accounted for by differences in gestational age at birth; however, the share of the mortality risk component increased from 21% to 32%. The gap increased after 2020, with sudden unexpected infant death showing the largest share increase of any cause.

**Meaning:**

These findings suggest that closing the infant mortality gap requires addressing both preterm birth disparities and growing differences in within–gestational age mortality risk, particularly for sudden unexpected infant death.

## Teaser

This cross-sectional study uses 22 years of mortality data to quantify gestational age distribution and mortality rates in infants born to Black mothers compared with infants born to White mothers.

## Introduction

The infant mortality rate (IMR) for infants born to non-Hispanic Black mothers in the US is more than twice that of infants born to non-Hispanic White mothers, a disparity that has persisted for decades.^1^ After years of decrease, the gap in Black and White IMR increased after 2020, as Black infant mortality increased while White infant mortality remained stable.^2^ Recent trend analyses have identified sudden unexpected infant death (SUID) as a notable exception to the long-term declines, with SUID rates increasing for the non-Hispanic Black population and the Black and White SUID disparity ratio increasing from 2.2 in 2017 to 2.8 in 2020.^2,3^

The IMR for infants born to non-Hispanic Black mothers is higher for 2 reasons. First, these infants are more likely to be born preterm, which increases the risk of dying in the first year—an age composition effect. Second, these infants face higher mortality risk within the same gestational age categories, reflecting a rate effect.^4,5^ To quantify the share of the age composition and rate effects, we applied the Kitagawa method,^6^ a demographic decomposition technique, to 22 years of infant mortality data. We also investigated the main causes of death to characterize the recent increase in the gap in Black and White IMR.

## Methods

This cross-sectional study was determined by the University of Colorado Institutional Review Board to not involve human participants. The board waived the requirement for informed consent because the study used existing data that did not contain identifiable private information. This study follows the Strengthening the Reporting of Observational Studies in Epidemiology (STROBE) reporting guideline for cross-sectional studies.

Data were obtained from publicly available Period Linked Birth/Infant Death Records from the National Center for Health Statistics from January 1, 2003, through December 31, 2024.^7^ These files link each infant death certificate to its corresponding birth certificate, covering approximately 99% of infant deaths. The analysis was restricted to births to non-Hispanic Black mothers and non-Hispanic White mothers with known gestational age. These racial and ethnic categories are produced by the National Center for Health Statistics and were drawn from birth certificate information. Records with implausible combinations of birth weight and gestational age were excluded using the inclusion criteria described by Alexander et al^8^ (eAppendix 1 in Supplement 1). Gestational age was classified into 7 categories: extremely preterm (<28 weeks), very preterm (28-31 weeks), moderately preterm (32-33 weeks), late preterm (34-36 weeks), early term (37-38 weeks), full or late term (39-41 weeks), and postterm (≥42 weeks).

### Statistical Analysis

The Black and White IMR difference was decomposed using the Kitagawa method,^6^ to partition the gap into an age composition effect (differences in the gestational age distribution) and a rate effect (differences in mortality within gestational age groups). We also performed this decomposition on each cause of death separately. To do so, we used *International Classification of Disease, Tenth Revision *(*ICD-10*) codes to group deaths into the top 13 top categories by underlying cause, with remaining deaths classified as other. By construction, summing the cause-specific composition effects recovers the overall composition effect, and similarly for rate effects. Full details and formulas for the decomposition are provided in eAppendix 2 in Supplement 1. We obtained 95% CIs for all rates and decomposition results using a Monte Carlo procedure (1000 iterations) that resampled cell-level death counts from a Poisson distribution, with the mean equal to the observed deaths, recomputed the decomposition, and provided the 2.5% and 97.5% quantiles. Sensitivity analyses were conducted using different gestational age classifications as well as a cause of death decomposition that split the combined SUID category into its 3 *ICD-10* components (eAppendix 3 and eTable 4 in Supplement 1).

Analyses were conducted in R, version 4.5 (R Project for Statistical Computing).^9^ Reproducible code and data are available in eAppendix 4 in Supplement 1. Statistical significance was defined as 95% CIs excluding 1.00.

## Results

**Figure 1.**
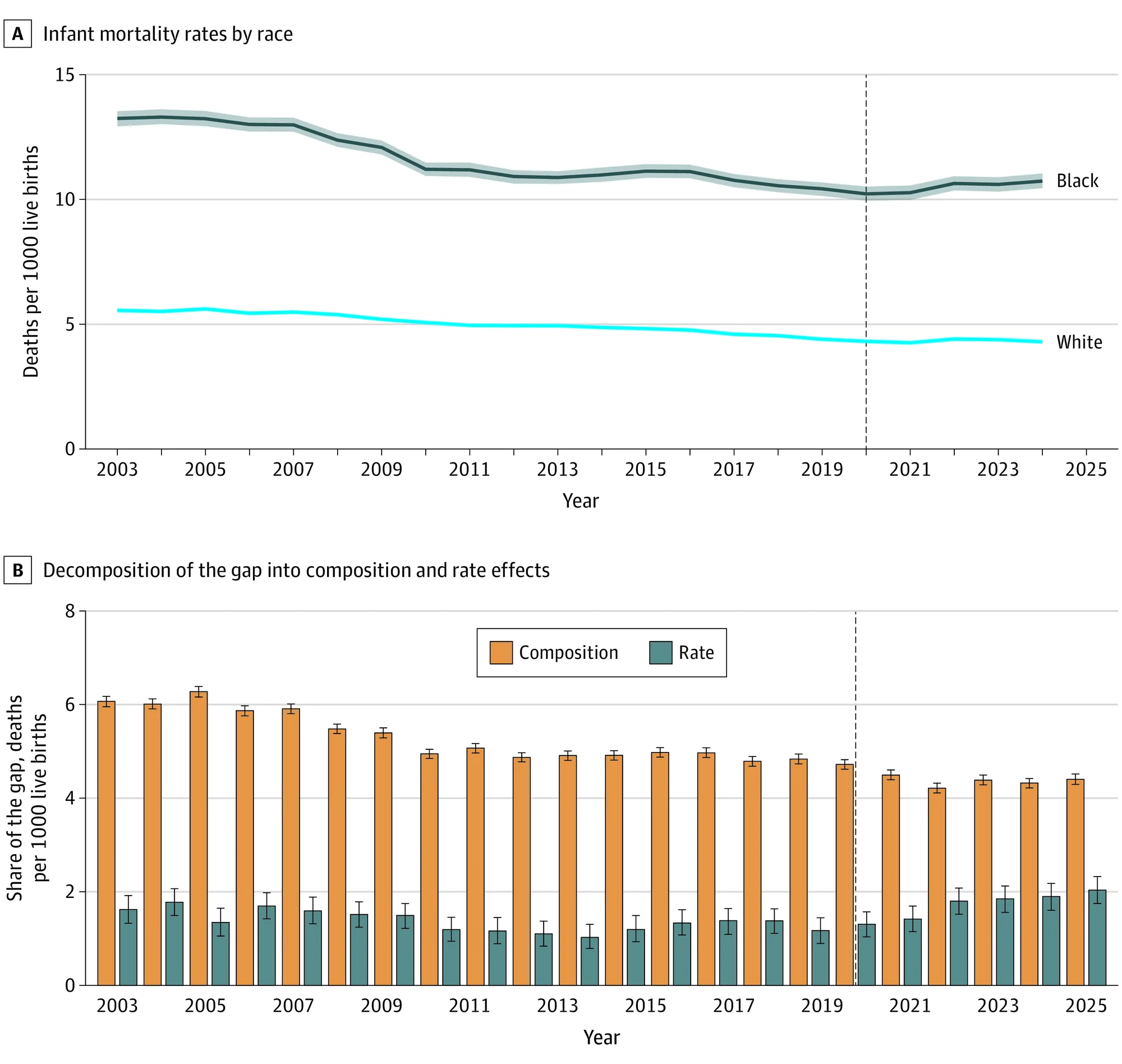
Line Graph of Infant Mortality by Race and Bar Graphs of Decomposition of Composition and Rate Effects, 2003-2024 A, Shaded regions of the line graph indicate 95% CIs; dashed line, 2020. B, Composition effect represents differences in gestational age distribution; rate effect, differences in within–gestational age mortality; error bars, 95% CI; dashed line, 2020.

There were 57 870 726 live births and 366 384 infant deaths in the non-Hispanic Black (141 651 [38.7%] of all deaths) and non-Hispanic White (224 733 [61.2% of all deaths) populations from January 1, 2003, to December 31, 2024 (eTable 1 in Supplement 1). Infants born to non-Hispanic Black mothers were more often born preterm (<37 weeks) compared with non-Hispanic White mothers (16.9% vs 10.5% over the study period). The Black IMR declined from 13.2 (95% CI, 12.9-13.5) to 10.7 (95% CI, 10.5-11.0) deaths per 1000 live births between 2003 and 2024 (19% reduction); the White IMR declined from 5.6 (95% CI, 5.5-5.7) to 4.3 (95% CI, 4.2-4.4) deaths per 1000 live births (23% reduction). The absolute Black and White infant mortality rate gap decreased from 7.7 to 6.0 deaths per 1000 live births between 2003 and 2019 but increased to 6.4 (95% CI, 6.1-6.8) by 2024, as Black infant mortality increased while White infant mortality continued to decline (Figure 1A).

In every year of the study period, the gestational age composition effect accounted for the majority of the gap in Black and White IMR. However, the mortality rate effect increased as a share of the gap, from 21% (95% CI, 18%-24%) in 2003 to 32% (95% CI, 28%-35%) in 2024. This shift increased beginning in 2020: between 2020 and 2024, the share of the rate effect increased from 1.42 (95% CI, 1.15-1.69) to 2.04 (95% CI, 1.75-2.33) deaths per 1000 live births, while the share of the age composition effect declined from 4.49 (95% CI, 4.39-4.60) to 4.40 (95% CI, 4.29-4.52) deaths per 1000 live births (Figure 1B). Sensitivity analyses showed similar results using more granular gestational age categories (eTable 4 in Supplement 1). Across study years, the earliest gestational age category (<28 weeks) accounted for over half the overall gap in IMR (in 2003, 5.29 [95% CI, 5.06-5.51] deaths per 1000 live births of a total gap of 7.7; in 2024, 3.51 [95% CI 3.31-3.72] deaths per 1000 live births of a total gap of 6.4) (eTables 2 and 3 in Supplement 1).

The Table shows cause-specific decomposition for 2024. SUID was the largest single cause contributor to the IMR gap (1.56 [95% CI, 1.42-1.71] deaths per 1000 live births). SUID deaths include those from sudden infant death syndrome, accidental suffocation and strangulation in bed, and other causes that remain unexplained even after investigation. Its contribution was due almost entirely to the rate effect (1.44 [95% CI, 1.30-1.59 of 1.56] deaths per 1000 live births). Short gestation or low birth weight was the second-largest contributor (1.44 [95% CI, 1.30-1.57] deaths per 1000 live births), but this was primarily an age composition effect.

**Table.** Cause-Specific Decomposition of the Gaps in Black and White IMR, With Kitagawa Decomposition for 2024

Cause of death	IMR gap, deaths per 1000 live births (95% CI)	Composition effect, deaths per 1000 live births (95% CI)^a^	Rate effect, deaths per 1000 live births (95% CI)^b^	Rate effect, %^c^
All causes	6.44 (6.14 to 6.75)	4.40 (4.29 to 4.52)	2.04 (1.75 to 2.33)	32
Sudden unexpected infant death	1.56 (1.42 to 1.71)	0.12 (0.10 to 0.14)	1.44 (1.30 to 1.59)	92
Short gestation or LBW	1.44 (1.30 to 1.57)	1.25 (1.19 to 1.30)	0.19 (0.07 to 0.29)	13
Other^d^	1.09 (0.95 to 1.23)	1.01 (0.96 to 1.06)	0.07 (−0.06 to 0.20)	7
Infection	0.52 (0.44 to 0.60)	0.35 (0.32 to 0.38)	0.17 (0.10 to 0.23)	32
Maternal complication	0.45 (0.37 to 0.52)	0.44 (0.41 to 0.47)	0.01 (−0.05 to 0.07)	1
Congenital malformation	0.42 (0.30 to 0.53)	0.44 (0.41 to 0.47)	−0.01 (−0.13 to 0.10)	−3
Respiratory distress	0.18 (0.14 to 0.24)	0.20 (0.18 to 0.22)	−0.02 (−0.06 to 0.03)	−9
Placenta, cord, or membranes	0.17 (0.11 to 0.22)	0.19 (0.17 to 0.21)	−0.02 (−0.07 to 0.02)	−15
Necrotizing enterocolitis	0.14 (0.10 to 0.18)	0.12 (0.10 to 0.13)	0.02 (−0.01 to 0.06)	17
Circulatory disease	0.13 (0.09 to 0.18)	0.06 (0.04 to 0.07)	0.08 (0.04 to 0.12)	59
Hypoxia and birth asphyxia	0.11 (0.06 to 0.15)	0.09 (0.08 to 0.11)	0.01 (−0.02 to 0.05)	14
Assault (homicide)	0.09 (0.06 to 0.13)	0.01 (0.00 to 0.01)	0.09 (0.05 to 0.12)	91
Unintentional injury	0.08 (0.05 to 0.11)	0.01 (0.00 to 0.02)	0.07 (0.04 to 0.11)	89
Hemorrhage	0.06 (0.03 to 0.10)	0.12 (0.10 to 0.13)	−0.05 (−0.09 to −0.02)	−89

Abbreviations: IMR, infant mortality rate; LBW, low birth weight.

^a^ Composition effect represents differences in gestational age distribution.

^b^ Rate effect indicates differences in within–gestational age mortality.

^c^ Rate percentage indicates the ratio of the rate to the overall gap.

^d^ Other represents any remaining cause of death.

**Figure 2.**
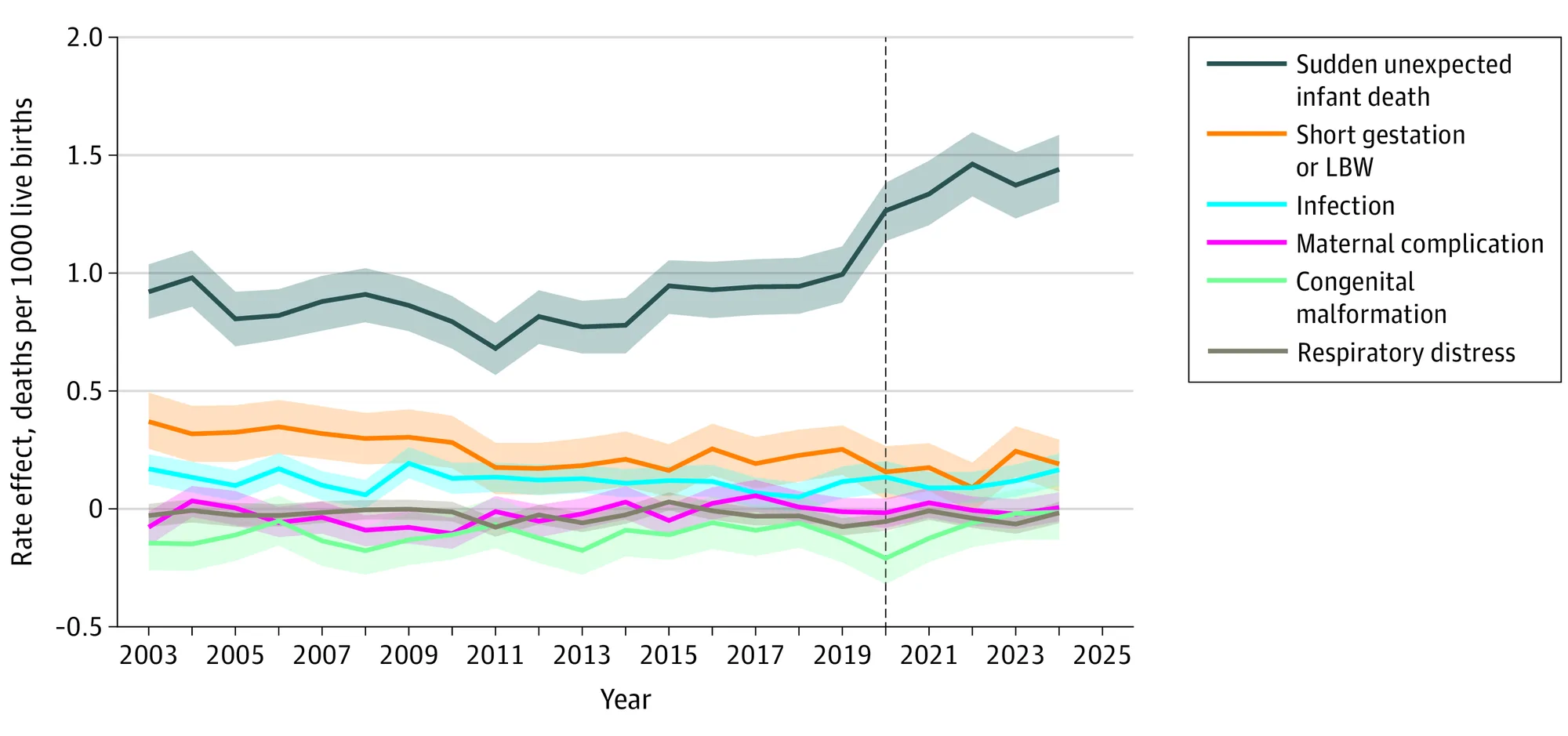
Line Graphs of Rate Effect (Within–Gestational Age Mortality Risk) in the Mortality Gap Between Infants Born to Black and White Mothers by Cause of Death, 2003-2024 LBW indicates low birth weight. Shaded regions indicate 95% CIs; dashed line, 2020.

Over the study period, SUID death had the largest rate effect of any cause, and this effect increased over time (Figure 2). From 2020 to 2024, the SUID rate effect increased from 1.26 (95% CI, 1.14-1.38) to 1.44 (95% CI, 1.30-1.59) deaths per 1000 live births, reflecting increasing disparities in within–gestational age SUID mortality rates rather than changes in the gestational age distribution. In the post–COVID-19 pandemic period (2020 and later), the share from SUID accounted for the largest increase of any single cause to the gap in IMR (0.18 deaths per 1000 live births; SUID gap in 2024, 1.56 [95% CI, 1.42-1.71] deaths per 1000 live births) (Figure 2). This increase accounted for 35% of the total net change (eFigure 1 in Supplement 1). Splitting the combined SUID category into its 3 *ICD-10* components showed that ill-defined or unspecified (R99) had the largest increase, although sudden infant death syndrome and accidental suffocation and strangulation in bed also showed increases since 2015 (eFigure 2 in Supplement 1).

## Discussion

In this analysis of 22 years of national linked birth-infant death data, we found that while gestational age composition differences remain the largest factor in the gap in Black and White IMR, the mortality risk component has increased and now accounts for nearly one-third of the gap. The increase in the IMR gap since 2020 was due in part to increasing SUID disparities.

The increasing rate effect indicates that within–gestational age mortality differences are increasing relative to composition differences. This finding suggests that even if preterm birth disparities were eliminated, a large mortality gap would remain. The concentration of a 2020 through 2024 increase in disparities in SUID warrants further investigation. This increase coincided with the COVID-19 pandemic, a respiratory pandemic, indicating a possible etiology for the large increase in SUID’s rate effect. The increase also paralleled racially disparate increases in maternal mortality during the pandemic.^10^ The dual burden of increasing racial disparities in both maternal and infant deaths underscores the interconnected nature of perinatal health inequities and the need for comprehensive strategies spanning the pregnancy to infancy continuum.

### Limitations

Here we examined only mothers who were non-Hispanic Black and non-Hispanic White, but infant mortality and particularly SUID death rates are also high among children born to non-Hispanic American Indian and Alaska Native and non-Hispanic women of more than 1 race.^11^ The Kitagawa method assumes additive effects and cannot capture interactions between composition and rate differences. Cause of death classification relies on death certificate coding, which may vary in accuracy across populations. Other demographic and socioeconomic characteristics, such as maternal age, parity, and educational attainment, could change differently over time in the non-Hispanic Black and non-Hispanic White populations. Future work could extend this analysis to investigate how these factors may contribute to the changing gap in IMR.

## Conclusions

In this cross-sectional study of infant mortality, gestational age composition differences continued to account for the largest share of the gap in Black and White IMR. However, the within–gestational age mortality risk component increased as a share of the overall gap from 2003 to 2024. The increased mortality risk component highlights the need for continued efforts to reduce preterm birth disparities, alongside targeted interventions addressing the increasing within–gestational age mortality risk differences, particularly for SUID.

## Notes

Supplement 1. **eAppendix 1.** Data exclusions **eTable 1.** Record exclusions, 2003-2024 **eFigure 1.** Change in each cause’s contribution to the Black-White IMR gap between 2020 and 2024 (deaths per 1,000 live births) **eFigure 2.** Rate effect (within-gestational-age mortality risk) contributions to the Black-White gap by the three ICD-10 components of Sudden Unexpected Infant Death, 2003-2024 **eTable 2.** Per-gestational-age decomposition of the Black-White infant mortality for 2003. **eTable 3. **Per-gestational-age decomposition of the Black-White infant mortality for 2024 **eAppendix 2**. Supplemental methods details **eAppendix 3. **Sensitivity analyses **eTable 4.** Comparison of 7-category (main) and 16-category gestational-age decomposition, 2024 **eAppendix 4.** Reproducible code and data

Supplement 2.
Data Sharing Statement
