# Supplementary material for: Decomposition of Black and White Infant Mortality Rates
Source: JAMA Netw Open. 2026 Sep 22;9(9):e2635321. doi: 10.1001/jamanetworkopen.2026.35321 (PMC13598697; doi:10.1001/jamanetworkopen.2026.35321)
Supplement: Supplementary file 1 [file jamanetwopen-e2635321-s001.pdf]

Supplementary Online Content

Alexander MJ, Alexander R, Root L. Decomposition of Black and White infant mortality rates. *JAMA Netw Open*. 2026;9(9):e2635321. doi:10.1001/jamanetworkopen.2026.35321

eAppendix 1. Data exclusions

eTable 1. Record exclusions, 2003-2024

eFigure 1. Change in each cause's contribution to the Black-White IMR gap between 2020 and 2024 (deaths per 1,000 live births)

eFigure 2. Rate effect (within-gestational-age mortality risk) contributions to the Black-White gap by the three ICD-10 components of Sudden Unexpected Infant Death, 2003-2024

eTable 2. Per-gestational-age decomposition of the Black-White infant mortality for 2003.

eTable 3. Per-gestational-age decomposition of the Black-White infant mortality for 2024

eAppendix 2. Supplemental methods details

eAppendix 3. Sensitivity analyses

eTable 4. Comparison of 7-category (main) and 16-category gestational-age decomposition, 2024

eAppendix 4. Reproducible code and data

This supplementary material has been provided by the authors to give readers additional information about their work.

© 2026 Alexander MJ et al. *JAMA Network Open*.

eAppendix 1. Data exclusions

Exclusion criteria are applied sequentially: maternal race/ethnicity outside non-Hispanic Black or non-Hispanic White, then missing gestational age, then implausible birthweight-for-gestational-age combinations per Alexander et al.[8] Table 1. Of the 202,450 live births excluded on the last criterion, 191,513 had a birthweight above the plausible range for the recorded gestational age, 4,632 below it, and 6,305 were missing birthweight.

eTable 1. Record exclusions, 2003-2024

Record type	Total records	Excluded: race/ethnicity	Excluded: unknown GA	Excluded: implausible birthweight/GA	Included in analysis
Live births (denominator)	86,797,383	28,632,271	91,936	202,450	57,870,726
Infant deaths (numerator)	522,169	148,274	3,705	3,806	366,384

© 2026 Alexander MJ et al. *JAMA Network Open*.

eFigure 1. Change in each cause's contribution to the Black-White IMR gap between 2020 and 2024 (deaths per 1,000 live births). SUID showed the largest increase, while three causes saw decreased contributions.

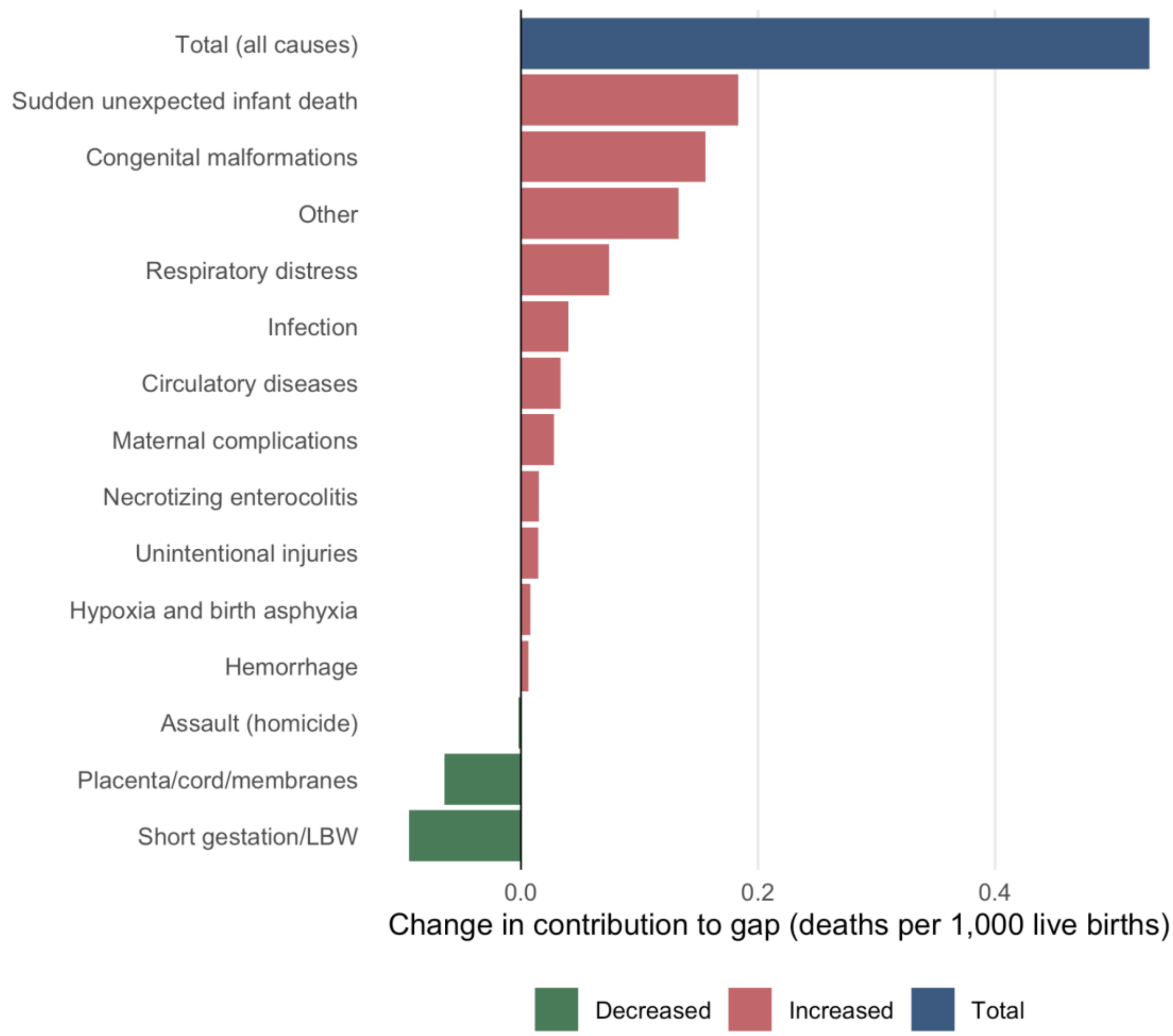

© 2026 Alexander MJ et al. *JAMA Network Open*.

eFigure 2. Rate effect (within-gestational-age mortality risk) contributions to the Black-White gap by the three ICD-10 components of Sudden Unexpected Infant Death, 2003-2024. Shaded bands show 95% confidence intervals

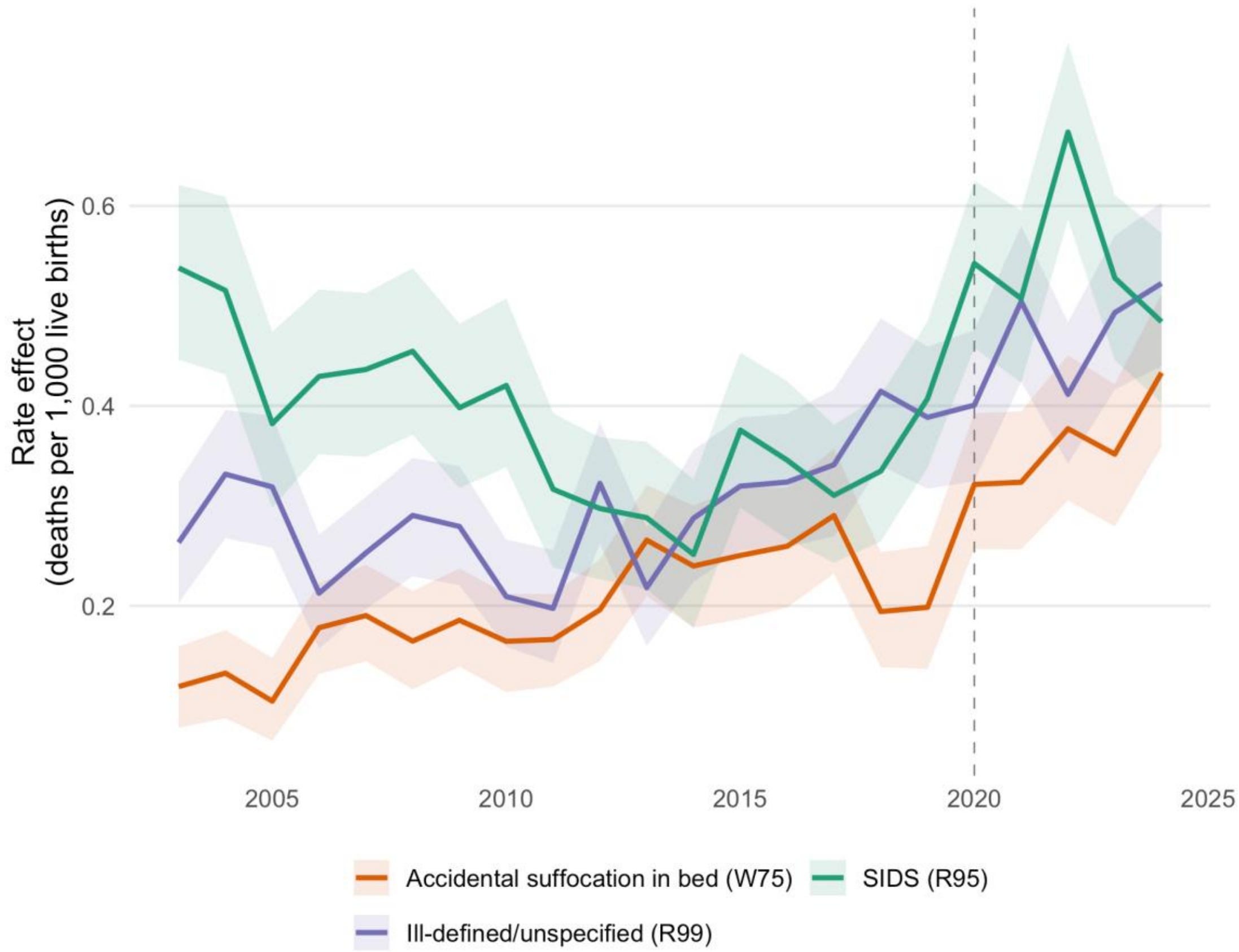

© 2026 Alexander MJ et al. *JAMA Network Open*.

eTable 2. Per-gestational-age decomposition of the Black-White infant mortality for 2003. Columns are: birth proportion by race; infant mortality rate per 1,000 by race; per-GA composition contribution; rate; total = composition + rate. Numbers in parentheses are 95% Monte Carlo confidence intervals. Summing the per-GA total contributions reproduces the overall.

Gestational age	Black prop.	White prop.	Black IMR	White IMR	Composition	Rate	Total
<28 weeks	0.017	0.005	438.21	436.98	5.27 (5.17 to 5.37)	0.01 (-0.19 to 0.21)	5.29 (5.06 to 5.51)
28-31 weeks	0.019	0.009	57.26	53.07	0.56 (0.53 to 0.59)	0.06 (-0.02 to 0.14)	0.62 (0.53 to 0.71)
32-33 weeks	0.023	0.013	17.45	16.74	0.17 (0.16 to 0.19)	0.01 (-0.04 to 0.06)	0.19 (0.13 to 0.24)
34-36 weeks	0.114	0.083	8.14	6.82	0.23 (0.22 to 0.25)	0.13 (0.05 to 0.21)	0.37 (0.28 to 0.45)
37-38 weeks	0.278	0.273	4.54	2.87	0.02 (0.02 to 0.02)	0.46 (0.36 to 0.55)	0.48 (0.38 to 0.57)
39-41 weeks	0.486	0.553	3.49	1.92	-0.18 (-0.19 to -0.17)	0.82 (0.69 to 0.94)	0.64 (0.52 to 0.75)
42+ weeks	0.061	0.063	4.67	2.60	-0.01 (-0.01 to -0.01)	0.13 (0.08 to 0.18)	0.12 (0.08 to 0.17)

© 2026 Alexander MJ et al. *JAMA Network Open*.

eTable 3. Per-gestational-age decomposition of the Black-White infant mortality for 2024. Columns are: birth proportion by race; infant mortality rate per 1,000 by race; per-GA composition contribution; rate; total = composition + rate. Numbers in parentheses are 95% Monte Carlo confidence intervals. Summing the per-GA total contributions reproduces the overall.

Gestational age	Black prop.	White prop.	Black IMR	White IMR	Composition	Rate	Total
<28 weeks	0.014	0.004	355.54	364.34	3.59 (3.49 to 3.70)	-0.08 (-0.26 to 0.10)	3.51 (3.31 to 3.72)
28-31 weeks	0.018	0.008	44.39	44.78	0.44 (0.41 to 0.47)	-0.00 (-0.08 to 0.07)	0.44 (0.35 to 0.53)
32-33 weeks	0.023	0.012	18.90	15.29	0.18 (0.16 to 0.19)	0.06 (0.01 to 0.12)	0.24 (0.18 to 0.31)
34-36 weeks	0.115	0.079	9.51	6.82	0.30 (0.28 to 0.31)	0.26 (0.17 to 0.35)	0.56 (0.46 to 0.66)
37-38 weeks	0.313	0.268	5.02	2.74	0.17 (0.16 to 0.18)	0.66 (0.56 to 0.78)	0.83 (0.72 to 0.95)
39-41 weeks	0.467	0.576	3.49	1.47	-0.27 (-0.28 to -0.26)	1.05 (0.93 to 1.19)	0.78 (0.67 to 0.91)
42+ weeks	0.050	0.052	3.93	2.41	-0.01 (-0.01 to -0.00)	0.08 (0.04 to 0.12)	0.07 (0.03 to 0.12)

© 2026 Alexander MJ et al. *JAMA Network Open*.

eAppendix 2. Supplemental methods details

Kitagawa decomposition method

The Kitagawa decomposition partitions the difference between two aggregate rates into two components: one attributable to differences in the composition of subgroups (here, gestational age groups) and one attributable to differences in subgroup-specific rates.

Let R_B and R_W denote the overall IMR for Black and White infants, respectively. Each overall rate is a weighted average of gestational-age-specific mortality rates:

$$R_r = \sum_i p_{r,i} \ M_{r,i}$$

where $p_{r,i}$ is the proportion of births in gestational age group i for race r, and $M_{r,i}$ is the IMR within group i for race r.

The Kitagawa decomposition expresses the total difference as:

$$R_B - R_W = \underbrace{\sum_i \left(p_{B,i} - p_{W,i}\right) \frac{M_{B,i} + M_{W,i}}{2}}_{\text{Age composition effect}} + \underbrace{\sum_i \left(M_{B,i} - M_{W,i}\right) \frac{p_{B,i} + p_{W,i}}{2}}_{\text{Rate effect}}$$

The age composition effect captures how much of the IMR gap is due to Black infants being more likely to be born at gestational ages where mortality is high (e.g., very preterm). The rate effect captures how much is due to Black infants experiencing higher mortality than White infants within the same gestational age groups.

Cause-specific extension

We extended this decomposition to individual causes of death. For each cause j, let $M_{r,i,j}$ denote the cause-specific mortality rate in gestational age group i for race r. The cause-specific decomposition is:

$$\text{Gap}_j = \underbrace{\sum_i \left(p_{B,i} - p_{W,i}\right) \frac{M_{B,i,j} + M_{W,i,j}}{2}}_{\text{Cause-specific composition effect}} + \underbrace{\sum_i \left(M_{B,i,j} - M_{W,i,j}\right) \frac{p_{B,i} + p_{W,i}}{2}}_{\text{Cause-specific rate effect}}$$

The birth proportions $p_{r,i}$ are the same as in the overall decomposition (they do not vary by cause). The cause-specific mortality rates sum to the overall rate within each gestational age group: $M_{r,i} = \sum_j M_{r,i,j}$. By construction, summing the cause-specific composition effects across all causes recovers the overall composition effect, and similarly for the rate effects:

$$\sum_j \text{Gap}_j = R_B - R_W$$

© 2026 Alexander MJ et al. *JAMA Network Open*.

This additive property allows us to identify which causes of death contribute most to the overall gap and to track how those contributions change over time.

Monte Carlo confidence intervals

We obtained 95% confidence intervals around all rates, decomposition components, and cause-specific contributions using a parametric bootstrap. For each cell defined by year × race × gestational-age group (and × cause of death for the cause-specific decomposition), we treated the observed death count d as Poisson-distributed with mean equal to the observed count and simulated a replacement count $d^{(k)} \sim \text{Poisson}(d)$. Live births in each cell were treated as fixed. From the simulated death counts we recomputed cell-level mortality rates ($d^{(k)}$/births $\times$ 1000), the overall race-specific IMRs, and the Kitagawa decomposition components using the formulas above. We repeated the procedure $K = 1000$ times and report the 2.5% and 97.5% quantiles of each derived quantity across simulations as the 95% confidence interval.

These intervals are model-based. They summarize the variation implied by treating cell-level death counts as Poisson random variables, conditional on the observed live births and cause-of-death classifications. Because the linked files capture nearly all infant deaths, the intervals reflect random variation in small counts rather than finite-population sampling error. The all-cause and cause-specific decompositions are simulated separately, so the cause-specific intervals are not jointly propagated with the all-cause intervals and need not sum to them. For rare causes and sparse gestational-age cells, many cells have zero or few deaths, where Poisson resampling introduces little variation and the resulting intervals may understate uncertainty.

© 2026 Alexander MJ et al. *JAMA Network Open*.

eAppendix 3. Sensitivity analyses

The 7-category scheme (the main analysis) uses <28, 28-31, 32-33, 34-36, 37-38, 39-41, and 42+ weeks. The 16-category scheme uses <28 weeks, each completed week from 28 to 41 individually, and 42+ weeks. Both schemes recover the same total Black-White gap by construction; only the apportionment between composition and rate effects differs, with finer stratification shifting a slightly larger share to the composition effect.

eTable 4. Comparison of 7-category (main) and 16-category gestational-age decomposition, 2024

GA scheme	Total gap	Composition effect	Rate effect	Composition %	Rate %
7-category (main)	6.44	4.40	2.04	68%	32%
16-category	6.44	4.45	1.99	69%	31%

eAppendix 4. Reproducible code and data

We used publicly available NCHS Linked Birth-Infant Death Data from the National Center for Health Statistics, National Vital Statistics System for the years 2003 to 2024. The data are available at https://www.cdc.gov/nchs/data_access/vitalstatsonline.htm. The variables used were Year of birth, mother's race, mother's Hispanic origin, mother's age, gestational age (weeks), infant death indicator.

Reproducible code is available at: https://github.com/MJAlexander/imr-decomposition.

© 2026 Alexander MJ et al. *JAMA Network Open*.
