# Supplementary material for: Decomposition of Black and White Infant Mortality Rates
Source: JAMA Netw Open. 2026 Sep 22;9(9):e2635321. doi: 10.1001/jamanetworkopen.2026.35321 (PMC13598697; doi:10.1001/jamanetworkopen.2026.35321)
Supplement: Supplementary file 2 [file jamanetwopen-e2635321-s002.pdf]

Data Sharing Statement

Alexander. Decomposition of Black and White Infant Mortality Rates. *JAMA Netw Open*. Published September 22, 2026. doi:10.1001/jamanetworkopen.2026.35321

Data
Data available: Yes
Data types: Deidentified participant data
How to access data: https://www.cdc.gov/nchs/data_access/vitalstatsonline.htm
When available: With publication

Supporting Documents
Document types: Statistical/analytic code
How to access documents: https://github.com/MJAlexander/imr-decomposition
When available: With publication

Additional Information
Who can access the data: Data are publicly available
Types of analyses: Data are publicly available
Mechanisms of data availability: Data are publicly available
